# Retinal pigment epithelial atrophy over polypoidal choroidal vasculopathy lesions during ranibizumab monotherapy

**DOI:** 10.1186/s12886-016-0237-x

**Published:** 2016-05-16

**Authors:** Taiichi Hikichi, Hirokuni Kitamei, Shoko Shioya

**Affiliations:** Hikichi Eye Clinic, Kita-7 Nishi-5 7-1 Kita-Sky-Bild 14F, Kita-ku, Sapporo 060-0807 Japan; Ohtsuka Eye Hospital, Sapporo, Japan

**Keywords:** Polypoidal choroidal vasculopathy, Ranibizumab, Retinal pigment epithelial atrophy

## Abstract

**Background:**

To evaluate the quantitative changes of retinal pigment epithelial (RPE) atrophy during 3-year follow-up period of ranibizumab monotherapy for polypoidal choroidal vasculopathy (PCV).

**Methods:**

We retrospectively reviewed consecutive 100 Japanese patients with unilateral symptomatic treatment-naïve PCV who received ranibizumab monotherapy for 3 years. Color fundus photography, spectral-domain optical coherence tomography, and fundus autofluorescence were evaluated for RPE atrophy. Multiple regression analysis was performed to investigate the predictive factors found during univariate analysis to identify an association with increased RPE atrophic areas. RPE atrophic areas overlapping PCV lesions were measured.

**Results:**

The mean (standard deviation) number of injections was 11.4 (4.50). RPE atrophic area enlarged to 2.91 (5.41 mm^2^) 3 years after the first injection from 1.22 (1.72 mm^2^) at baseline, which differed significantly (*P* = 0.012). Multiple regression analysis showed that larger PCV lesions and larger RPE atrophic areas at baseline were associated with increased RPE atrophic areas. RPE atrophic area overlapping the baseline PCV lesions significantly increased during 3-year follow-up period, whereas RPE atrophic area not overlapping the baseline PCV lesions did not increase significantly.

**Conclusion:**

RPE atrophy progresses in eyes with PCV during ranibizumab monotherapy and the tendency for development of RPE atrophy within the PCV lesions.

## Background

Polypoidal choroidal vasculopathy (PCV) was classically defined based on indocyanine green angiography (ICGA) and clinically seen as orange “polyplike” structures [1, 2], which are present at the terminal of a network of branching vessels. Pachychoroid pigment epitheliopathy is a recent newly described clinical entity characterized by a range of retinal pigment epithelial (RPE) abnormalities overlying the areas of choroidal thickening [3]. It is believed to reside within a spectrum of diseases including central serous chorioretinopathy, which involves choroidal congestion and choroidal hyperpermeability, resulting in focal RPE atrophy [3]. Long-standing “silent” pachychoroid pigment epitheliopathy may develop type 1 choroidal neovascularization (CNV), and ultimately PCV [4]. Progression of RPE atrophy is common in the natural history of PCV [5]. Polypoidal lesions and branching vascular networks were hypoautofluorescent on fundus autofluorescence (FAF) images, suggesting that neovascular lesion may directly affect overlying RPE cells in eyes with PCV [6, 7].

Vascular endothelial growth factor (VEGF) plays a critical role in both survival and maintenance of RPE integrity [8], and RPE-derived VEGF is an essential factor for maintenance of choriocapillaris [9, 10]. The SEVEN-UP study [11, 12] reported that most eyes with age-related macular degeneration (AMD) require anti-VEGF treatment over a long period, and the constant neutralization of VEGF may cause RPE atrophy and choriocapillary atrophy threatening visual conditions. The Comparison of Age-related Macular Degeneration Treatments Trials (CATT) [13, 14] also reported that the incidence of geographic atrophy (GA) was 18.3 % during 2 years of anti-VEGF treatment.

However, little information of RPE atrophy during anti-VEGF treatment is available about Asian patients. Recently, Kuroda and associates [15] studied Japanese patients with treatment-naïve neovascular AMD treated with ranibizumab (Lucentis, Genentech Inc., South San Francisco, CA), and concluded that RPE atrophy developed during ranibizumab treatment less frequently in Japanese patients compared with Caucasian patients and RPE atrophy progression rate was significantly faster in typical AMD than in PCV.

In the current study, we especially focused on the qualitative changes in RPE atrophy during 3-year follow-up period of ranibizumab monotherapy in eyes with PCV and investigated the characteristics of changing RPE atrophy.

## Methods

The current research followed the tenets of the Declaration of Helsinki, and all patients provided informed consent after explanation of the study protocol. The institutional review board at Ohtsuka Eye Hospital approved this retrospective study.

Data were collected retrospectively from consecutive Japanese patients with newly diagnosed, symptomatic, unilateral, treatment-naïve PCV at the Ohtsuka Eye Hospital who were treated with ranibizumab monotherapy and followed for 3 years. Treatment consisted of 0.5-mg intravitreal ranibizumab injection (IVR) for 3 months followed by as-needed reinjections based on monthly examinations between June 2010 and December 2012. There were no exclusion criteria for baseline VA or lesion size; however, eyes were excluded if there was a massive subretinal hemorrhage or subpigment epithelial hemorrhage that obscured the FAF findings, a spherical equivalent of -6.0 diopters or less or chorioretinal atrophic changes secondary to other disorders, and a history of treatment with anti-VEGF therapy and/or photodynamic therapy in the fellow eye.

PCV was defined as the presence of one or multiple focal areas of hyperfluorescence from the inner choroidal vessels within the first 6 min after injection of indocyanine green with or without an associated branching vascular network [16]. All patients underwent comprehensive ocular examinations including measurement of the best-corrected visual acuity (VA) using a Landolt ring chart and intraocular pressure, indirect ophtamoscopy, slit-lamp biomicroscopy with a preset lens, color fundus photography, digital simultaneous fluorescein angiography and ICGA and FAF imaging with a confocal scanning laser ophthalmoscope (HRA-2, Heidelberg Engineering Inc., Heidelberg, Germany), and spectral-domain optical coherence tomography (OCT) (Spectralis, Heidelberg Engineering Inc.), at the baseline examination. Generally, the ranibizumab first was injected on the same day as the baseline examination after the patients provided informed consent. FAF imaging was performed at baseline, 1 month after the three monthly injections, 12 months after the first IVR, and then every 6 months during the follow-up period.

The FAF images were acquired using a 488-nm excitation wave length, and emitted fluorescence signals were detected between 500 to 700 nm using the high-speed mode. The field of view was set at 30 × 30° with digital image resolution of 768 × 768 pixels and centered on the macula. The dramatic decrease of the FAF signal in the area of RPE atrophy compared with the nonatrophic retinal areas is used by the Region Finder, which can semiautomatically quantify atrophic areas and allows FAF images to be processed directly [17], for segmentation of the atrophic areas. Retinal blood vessels with intensities similar to those of the atrophic areas were excluded using the retinal blood vessel detection tool. Because of luteal pigment, blue-light FAF intensities typically decrease in the fovea. Although atrophic areas exhibit an even lower FAF intensity than the central macula, foveal involvement can be challenging to identify. Therefore, readers were instructed to use constraints while using the corresponding blue reflectance (488 nm) and near-infrared reflectance (820 nm) images to improve visualization of the foveal lesion boundary. Hypofluorescent signals on FAF images also are seen with fresh hemorrhages, lipid deposits, dense hyperpigmentation, RPE tears, and some forms of hard drusen. To eliminate such lesions, fundus photos and OCT findings were used as references. For each visit, the size of the total atrophic area was measured using the semiautomatic procedure. Two examiners (TH, HK) measured the RPE atrophic areas on all FAF images, and an intermediate value was adopted as the value of RPE atrophic area. To determine the inter-examiner (T.H., H.K.) reliability in the measurement of the RPE atrophic areas, the interclass correlation was calculated. Intra-examiner reliability also was evaluated by the intraclass correlation, which was calculated from the measurements of the RPE atrophic areas on 50 randomly selected cases on different two days by one examiner (T.H.).

Several possible factors were considered that may affect changes in the RPE atrophic area during 3-year ranibizumab therapy. The data recorded were age, gender, baseline VA, baseline areas of PCV lesions including polypoidal lesions and branching vascular networks, baseline location of PCV lesion, baseline RPE atrophic area in the treated and fellow eyes, presence of hemorrhages exceeding two optic disc areas at baseline, presence of a serous or hemorrhagic RPE detachment exceeding one optic disc area at baseline, number of IVR, and presence of intraretinal and subretinal fluid at baseline. To investigate the association between the change of RPE atrophic area during ranibizumab therapy and the factors measured on a continuous scale (i.e., age, VA, PCV lesion area, RPE atrophic area, and number of injections), simple regression analysis was performed (Table 2). In the categorical factors, the differences in change of RPE atrophic area during ranibizumab therapy between two mean values of categories or among more than three categories were analyzed using the Student’s *t*-test or one-way analysis of variance test, respectively (Table 2). To investigate the predictive factors of increased RPE atrophic area during 3-year ranibizumab therapy, multiple regression analysis was performed using the factors that achieved *P* < 0.2 in the univariate analysis. The logarithm of the minimum angle of resolution (logMAR) VA converted from the decimal VA was used to analyze the VA. All data analyses were performed using SAS version 9.3 software (SAS Inc., Cary, NC).

To investigate the tendency that RPE atrophy may progress over PCV lesions including polypoidal lesions and branching vascular networks during ranibizumab therapy, areas of RPE atrophy overlapping baseline PCV lesions were measured. The PCV lesions on the baseline ICGA images and areas of RPE atrophy on the FAF images were highlighted. Using an optic disc and retinal vessels as landmarks to superimpose the two images, the two images then were overlapped on the ImageJ software (version 1.48, National Institutes of Health, Bethesda, MD) and the areas of RPE atrophy overlapping and not overlapping PCV lesions were calculated.

## Results

Table 1 shows the baseline clinical characteristics of 100 patients and 3-year results of ranibizumab therapy. The mean (standard deviation) logMAR VA improved significantly (*P* = 0.027) from 0.33 (0.36) to 0.23 (0.31). The mean number of injections including the initial three monthly injections administered during the follow-up period was 11.4 (4.5). Fourteen (14 %) patients participated in our previous study [18] in which prognostic factors of 2-year outcomes of ranibizumab therapy for PCV were investigated.

**Table 1:** Baseline characteristics and three-year outcomes during ranibizumab monotherapy [mean (SD)]

	All eyes (*n* = 100)	Baseline RPE atrophy in treated eye	
		Absent (*n* = 16)	Present (*n* = 84)
Age (years)	71.5 (9.5)	70.0 (8.9)	71.8 (9.7)
RPE atrophy area (mm^2^)			
Baseline	1.22 (1.72)	0	1.43 (1.80)
Three years after the first IVR	2.91 (5.41)	0.55 (0.35)	3.36 (5.79)
Change value	1.69 (5.04)	0.55 (0.35)	1.92 (5.47)
RPE atrophy area in fellow eyes (mm^2^)			
Baseline	0.93 (1.33)	0.02 (0.01)	1.09 (1.95)
Three years after the first IVR	1.14 (1.69)	0.24 (0.60)	1.31 (2.24)
Change value	0.22 (0.79)	0.22 (0.64)	0.22 (1.05)
Visual acuity (logMAR value)			
Baseline	0.33 (0.36)	0.31 (0.09)	0.33 (0.39)
Three years after the first IVR	0.23 (0.31)	0.19 (0.22)	0.25 (0.32)
Change value	0.10 (0.25)	0.12 (0.14)	0.08 (0.28)
PCV lesion area (mm^2^) at baseline	2.93 (1.60)	2.46 (0.99)	3.02 (1.68)
No. injections during three years	11.4 (4.50)	10.3 (3.51)	11.6 (4.82)

*SD* standard deviation, *RPE* retinal pigment epithelium, *logMAR* logarithm of the minimum angle of resolution, *IVR* intravitreal injection of ranibizumab, *PCV* polypoidal choroidal vasculopathy

**Fig. 1:**
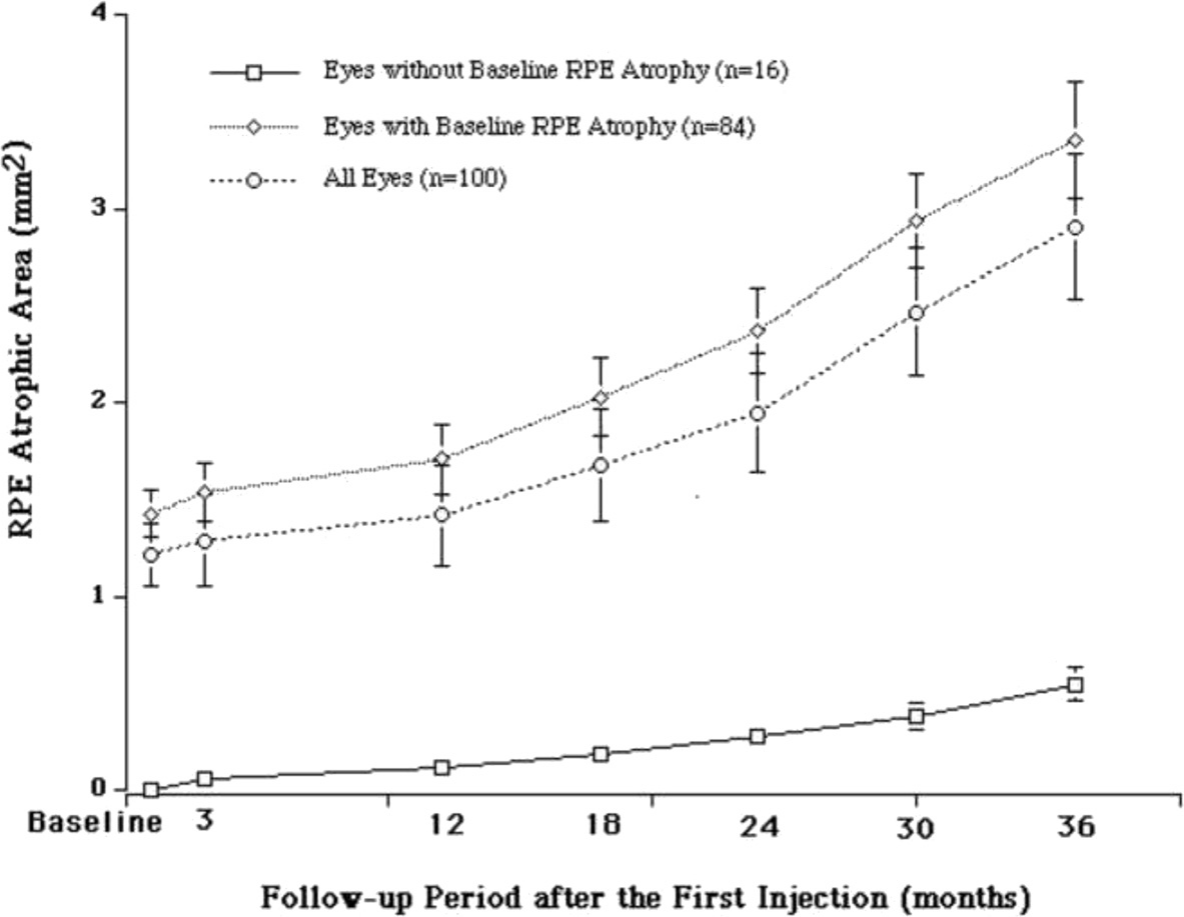
Mean area (mm^2^) of retinal pigment epithelial atrophy at various points during the follow-up period. RPE = retinal pigment epithelium. Error bars show standard error

RPE atrophy was found in 84 (84 %) eyes and not found in 16 (16 %) eyes at baseline. In all 100 eyes, RPE atrophic area progressed significantly (*P* = 0.012) from 1.22 (1.72 mm^2^) at baseline to 2.91 (5.41 mm^2^) 3 years after the first IVR (Fig. 1). Before using the Student’s *t*-test to compare the values between baseline and 3 years after the first IVR, a normal distribution of the data was confirmed using the Kolmogrov Smirnov test. In 84 eyes with RPE atrophy at baseline and 16 eyes without RPE atrophy at baseline, RPE atrophy area increased significantly (*P* = 0.026 and *P* = 0.001, respectively) (Fig. 1). In the fellow eyes, the RPE atrophic area slightly increased during 3-year follow-up period, which did not differ significantly. The rate by which the area increased annually in the treated eyes [0.56 (1.37 mm^2^)] was significantly (*P* = 0.001) faster compared to that in the fellow eyes [0.07 (0.28 mm^2^)].

Univariate analysis showed that advancing age, worse baseline VA, larger PCV lesions at baseline, and larger RPE atrophic areas at baseline in the treated eyes were the risk factors for increased RPE atrophic areas during ranibizumab therapy (Table 2). The number of IVR during 3-year follow-up period was not associated with increased RPE atrophic areas. Stepwise regression analysis showed that larger PCV lesions and larger RPE atrophic areas at baseline were associated significantly (adjusted *R*^*2*^ = 0.43, *P* = 0.001) with increased RPE atrophy areas during 3-year follow-up period of ranibizumab monotherapy (Table 3).

**Table 2:** Univariate analysis for the association between each factor and increased retinal pigment epithelial atrophic area (mm^2^) during the follow-up period of three years

	No.	Increased RPE atrophic area (mm^2^) in treated eyes [Mean (SD)]	*P* value
Age (years)			0.014*
≦69	42	1.21 (4.00)	
70-79	34	1.72 (3.98)	
≧80	24	2.10 (3.97)	
Gender			0.671†
Female	48	1.67 (4.25)	
Male	52	1.71 (4.96)	
Baseline decimal VA			0.024*
≧0.5	70	1.65 (5.08)	
0.2 - 0.4	20	2.44 (3.51)	
≦0.1	10	3.58 (3.15)	
PCV lesion area (DA)			0.001*
≦1	29	1.34 (3.79)	
> 1 to ≦2	39	1.45 (3.88)	
> 2 to 4	17	2.08 (3.92)	
> 4	15	2.57 (3.94)	
PED at baseline			0.576†
No	68	1.58 (4.33)	
Yes	32	1.83 (4.36)	
Hemorrhage at baseline			0.639†
No	78	1.60 (4.15)	
Yes	22	1.77 (3.85)	
Area of RPE atrophy at baseline (DA)			0.003*
Incident	16	0.55 (0.35)	
Prevalent	84	1.92 (5.47)	
≦0.5	44	1.53 (2.95)	
> 0.5 to 1	20	2.07 (3.12)	
> 1	20	2.59 (3.92)	
Area of RPE atrophy in fellow eye at baseline (DA)			0.967*
≦0.5	39	1.55 (3.41)	
> 0.5	61	1.77 (4.07)	
Location of PCV lesion			0.928†
Subfoveal	88	1.70 (5.24)	
Not subfoveal	12	1.65 (3.95)	
Intraretinal fluid at baseline			0.657‡
None	11	1.73 (3.46)	
Not subfoveal	6	2.08 (2.15)	
Subfovea	83	1.67 (4.64)	
Subretinal fluid at baseline			0.687‡
None	76	1.67 (3.31)	
Not subfoveal	6	1.72 (3.62)	
Subfovea	18	1.86 (3.93)	
Number of IVR during 3-year follow-up period			0.336*
1-5	8	1.69 (2.71)	
6-10	36	1.68 (3.86)	
11-15	42	1.70 (4.25)	
≧16	14	1.66 (2.25)	
Polypoidal lesions three years after the first IVR			0.871†
Detection	78	1.68 (4.45)	
No detection	22	1.72 (4.73)	
Decimal VA three years after the first IVR			0.016*
≧0.5	78	1.39 (4.83)	
0.2 - 0.4	15	2.44 (3.86)	
≦0.1	7	3.31 (2.29)	

*RPE* retinal pigment epithelium, *SD* standard deviation, *VA* visual acuity, *PCV* polypoidal choroidal vasculopathy, *DA* disc area, *PED* pigment epithelial detachment, *IVR* intravitreal ranibizumab injection

*, simple regression analysis; †, Student’s *t*-test; ‡, one-way analysis of variance test

**Table 3:** Stepwise regression analysis of risk factors for increased areas of retinal pigment epithelial atrophy during three-year follow-up period of ranibizumab monotherapy

Characteristic	Standardized regression coefficient	95 % confidence interval	*P* value
Baseline PCV area	0.32	0.23 to 0.40	0.001
Baseline RPE			
atrophic area	0.379	0.32 to 0.45	0.001

Larger PCV lesions and larger RPE atrophic areas at baseline are associated significantly (adjusted R^2^ = 0.43, *P* = 0.001) with increased RPE atrophic areas during ranibizumab therapy

*PCV* polypoidal choroidal vasculopathy, *RPE* retinal pigment epithelium

**Fig. 2:**
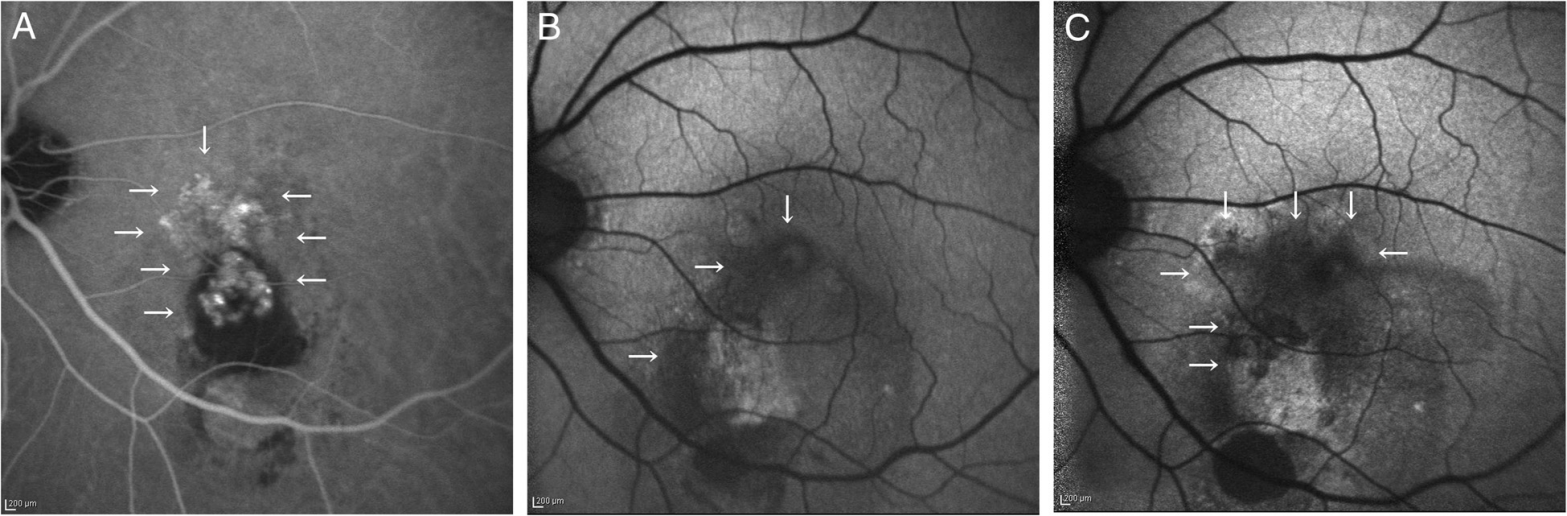
Progression of retinal pigment epithelial atrophy (RPE) over polypoidal choroidal vasculopathy (PCV) lesion during ranibizumab therapy. **a** An indocyanine green angiography (ICGA) image at baseline. *White arrows* indicate the area of PCV lesions including polypoidal lesions and branching vascular networks. **b** A fundus autofluorescence (FAF) image at baseline. *White arrows* indicate the area of RPE atrophy. **c** A native FAF image at month 36. *White arrows* indicate the area of RPE atrophy, which enlarges over PCV lesion compared to the baseline area

The RPE area overlapping the baseline PCV lesions significantly increased from 0.98 (1.87 mm^2^) to 2.53 (5.86 mm^2^) 3 years after the first IVR (*P* = 0.016), whereas the RPE area not overlapping the baseline PCV lesions changed from 0.21 (0.46 mm^2^) to 0.40 (0.77 mm^2^), which did not differ significantly (Fig. 2).

The baseline ICGA images showed polypoidal lesions in all 100 eyes. No polypoidal lesions were detected on ICGA images in 22 (22 %) eyes 3 years after the first IVR. Regarding the changes in the RPE atrophic area during the follow-up period, no apparent difference was found between eyes with and without resolution of polypoidal lesions (Table 4). Branching vascular networks remained in all eyes on ICGA images 3 years after the first IVR. In eyes with worse VA 3 years after the first IVR, increased RPE atrophic area significantly larger (*P* = 0.027) (Table 4).

**Table 4:** Correlation between Retinal Pigment Epithelial Atrophic Area Three years After the First ranibizumab injection and Resolution of Polypoidal lesions or Visual Acuity Three years After the First ranibizumab injection

		Increased RPE atrophic area (mm^2^)	
	No.	(Mean ± SD)	*P* Value
Polypoidal lesions three years after the first IVR			0.871
Detection	78	1.68 ± 4.45	
No detection	22	1.72 ± 4.73	
Decimal VA three years after the first IVR			0.027
≧0.5	78	1.39 ± 4.83	
0.2 - 0.4	15	2.44 ± 3.86	
≦0.1	7	3.31 ± 2.29	

*SD* standard deviation, *IVR* intravitreal ranibizumab injection; *VA* visual acuity

There was relatively good reproducibility (interclass correlation, 0.89; 95 % confidence interval, 0.79-0.96) between the two examiners who measured RPE atrophic areas. The intra-examiner agreement also was good (intraclass correlation, 0.94; 95 % confidence interval, 0.86-0.97).

During the follow-up period, no endophthalmitis, uveitis, lens damage, or prolonged intraocular pressure elevation occurred during the study period.

## Discussion

In the current study, we investigated the quantitative changes in RPE atrophy in eyes with PCV treated with ranibizumab through an as-needed protocol and found significant enlargement of RPE atrophy areas. Furthermore, larger PCV lesions and RPE atrophic areas at baseline predicted progressing RPE atrophic areas during ranibizumab therapy. Since larger areas have a longer border, and deterioration of RPE cells adjacent to RPE atrophic areas may occur, larger RPE atrophic areas at baseline may be correlated with the risk for progressing RPE atrophic areas during the follow-up period. Moreover, the most notable result of the current study was a tendency for progression of RPE atrophy over PCV lesions during ranibizumab therapy. This finding explains that larger PCV lesions at baseline predict progressing RPE atrophy.

Although the mean VA improved during 3-year follow-up of this study in spite of increased RPE atrophic area, worse VA 3 years after the first IVR correlated with larger area of RPE atrophy. Although clinical examination of AMD have indicated that the fovea tend to remain uninvolved by the RPE atrophic process until late in the disease course and follow-up period of anti-VEGF therapy [13, 14, 19], because RPE atrophy seems to progress over time [12], the long-term effect of these treatments on the central VA needs to be ascertained for the long term.

Although the current study did not directly address any causal relationship such as whether anti-VEGF treatment itself causes acceleration of RPE atrophy in PCV or whether development of RPE atrophy is part of the underlying disease progression of PCV, the results indicated that RPE atrophy tends to progress over PCV lesions and the total number of injections was not associated significantly with the quantitative amount of progressing RPE atrophy during ranibizumab therapy. Spectral-domain OCT images obtained from eyes with PCV showed that branching choroidal vascular networks and polypoidal lesions exist between the RPE and Bruch’s membrane and adhere to the back surface of the RPE layers [20]. Yamagishi et al. [7] described two characteristic patterns of hypoautofluorescence on FAF images compared with ICGA images, i.e., the well-demarcated confluent hypoautofluorescence corresponding to the polyp and heterogeneous granular hypoautofluorescence consistent with branching vascular networks on ICGA images, and suggested that in eyes with PCV, the presence of a neovascular lesion may directly affect the overlying RPE cells. Sato et al. [21] reported the “double layer sign” in eyes with PCV corresponding to the branching choroidal vascular networks and fluid accumulation between the RPE and Bruch’s membrane. Similar to the polypoidal lesions, the branching choroidal vascular networks seem to have an exudative property and may have a significant hemodynamic effect on the RPE layer. In addition, even without exudative changes, the presence of a branching vascular network adhering to the back surface of the RPE layers may be associated with RPE atrophy.

RPE-derived VEGF plays an essential role in maintenance of the choriocapillaris and cone photoreceptors [8, 10]. A study [10] of deletion of VEGF-A and VEGF isoforms in adult mice have suggested that sustained VEGF antagonism diminishes the trophic effects of RPE-produced VEGF, leading to exacerbation of RPE and choriocapillaris atrophy. Ford et al. [8] also reported that VEGF plays a critical role in survival and maintenance of RPE integrity and stated that potential undesired off-target effects should be considered with chronic use of anti-VEGF agents. Conversely, in the reports [13, 14] from the CATT Research Group, although a larger number of injections was associated with increased risk of GA development in the CATT study, once GA developed, the growth rate was not associated significantly with the number of injections. The reasons for this discrepancy were not discussed. In the current study, the total number of injections was not associated significantly with the quantitative amount of progressing RPE atrophy during ranibizumab therapy. Further clinical studies are necessary to confirm the relationship between the RPE damage and the number of anti-VEGF injections.

Resolution or persistence of polypoidal lesions that responded to ranibizumab therapy was not associated with the size of the increased area of RPE atrophy during the follow-up period in the current study. Yamagishi et al. [6] reported that at the follow-up examination, the baseline confluent and granular hypoautofluorescence of FAF was unchanged, even after complete regression of exudation and resolution of the polypoidal lesions. We think that since RPE atrophy tends to progress over PCV lesions including polypoidal lesions and branching vascular networks and areas of branching vascular networks are generally larger than those of PCV lesions, the effect of resolution or no resolution of the polypoidal lesions on the changes in RPE atrophy area may be relatively small in total RPE atrophic area.

## Conclusions

The limitations of the current study included its retrospective nature and the relatively small number of patients. Another limitation is that the current study did not directly address any causal relationship such as whether anti-VEGF treatment itself causes acceleration of RPE atrophy or whether development of RPE atrophy is part of the underlying disease progression of PCV. Despite these shortcomings, the study found progressing RPE atrophy in eyes with PCV during ranibizumab monotherapy and the tendency for development of RPE atrophy within the PCV lesions. This information may guide future treatment strategies for PCV, such as the need to protect the RPE cells to maintain the improved VA after the treatment over the long term.

### Ethics and content to participate

The current research followed the tenets of the Declaration of Helsinki, and all patients provided informed consent after explanation of the study protocol. The institutional review board at Ohtsuka Eye Hospital approved this retrospective study.

### Consent of publication

Informed consent for the publication of images relating to individual participants were obtained from the participants.

### Availability of data and materials

Since consent to publish was not obtained from all participants, the data will not be made available in order to protect the participants identity.

## Abbreviations

AMD: age-related macular degeneration
CNV: choroidal neovascularization
FAF: fundus autofluorescence
ICGA: indocyanine green angiography
IVR: intravitreal ranibizumab injection
logMAR: logarithm of the minimum angle of resolution
OCT: optical coherence tomography
PCV: polypoidal choroidal vasculopathy
RPE: Retinal pigment epithelium
SD: standard deviation
VA: visual acuity
VEGF: vascular endothelial growth factor
